# Asian hate speech detection on Twitter during COVID-19

**DOI:** 10.3389/frai.2022.932381

**Published:** 2022-08-15

**Authors:** Amir Toliyat, Sarah Ita Levitan, Zheng Peng, Ronak Etemadpour

**Affiliations:** ^1^Computer Science Program, Graduate Center, City University of New York, New York, NY, United States; ^2^Computer Science Program, Hunter College, City University of New York, New York, NY, United States; ^3^Computer Science Program, City College, City University of New York, New York, NY, United States

**Keywords:** Twitter, natural language processing, machine learning, COVID-19, Asian hate crime

## Abstract

Coronavirus disease 2019 (COVID-19) started in Wuhan, China, in late 2019, and after being utterly contagious in Asian countries, it rapidly spread to other countries. This disease caused governments worldwide to declare a public health crisis with severe measures taken to reduce the speed of the spread of the disease. This pandemic affected the lives of millions of people. Many citizens that lost their loved ones and jobs experienced a wide range of emotions, such as disbelief, shock, concerns about health, fear about food supplies, anxiety, and panic. All of the aforementioned phenomena led to the spread of racism and hate against Asians in western countries, especially in the United States. An analysis of official preliminary police data by the Center for the Study of Hate & Extremism at California State University shows that Anti-Asian hate crime in 16 of America's largest cities increased by 149% in 2020. In this study, we first chose a baseline of Americans' hate crimes against Asians on Twitter. Then we present an approach to balance the biased dataset and consequently improve the performance of tweet classification. We also have downloaded 10 million tweets through the Twitter API V-2. In this study, we have used a small portion of that, and we will use the entire dataset in the future study. In this article, three thousand tweets from our collected corpus are annotated by four annotators, including three Asian and one Asian-American. Using this data, we built predictive models of hate speech using various machine learning and deep learning methods. Our machine learning methods include Random Forest, K-nearest neighbors (KNN), Support Vector Machine (SVM), Extreme Gradient Boosting (XGBoost), Logistic Regression, Decision Tree, and Naive Bayes. Our Deep Learning models include Basic Long-Term Short-Term Memory (LSTM), Bidirectional LSTM, Bidirectional LSTM with Drop out, Convolution, and Bidirectional Encoder Representations from Transformers (BERT). We also adjusted our dataset by filtering tweets that were ambiguous to the annotators based on low Fleiss Kappa agreement between annotators. Our final result showed that Logistic Regression achieved the best statistical machine learning performance with an F1 score of 0.72, while BERT achieved the best performance of the deep learning models, with an F1-Score of 0.85.

## 1. Introduction

Coronavirus disease 2019 (COVID-19) is a disease caused by the coronavirus and, since 2019, has spread rapidly through the world, which created an international public health crisis. The impact of this previously unknown disease has been felt directly by its devastating symptoms and many deaths, but also indirectly in every aspect of life, severely curtailing normal activities such as shopping, working, socializing, and simply moving around (Chun et al., 2020). During the early months of the pandemic, when the disease was created and spread, the lack of a vaccine or treatment heightened the uncertainties about its health consequences. The number of confirmed patients and deaths had increased rapidly, and the disease spread daily into new areas. To control the spread of the disease, governments worldwide adopted robust measures to limit any non-essential social activities. These limitations caused more significant adverse impacts, such as border closings, airline shut downs, losing jobs, financial breakdowns, economic downturns toward depression, and supply chain disruptions, to name a few. These uncertainties and disturbances caused a substantial degree of anxiety, fear, and depression which was often channeled as hate toward Asians because Asians were perceived as the cause of all these misfortune (Chun et al., 2020). In this crisis, the targeted groups of Asians are specifically “the ethnic groups with origins in East Asia, Southeast Asia, or the Indian subcontinent” (CEN, 2021).

An analysis of hate crime events against Asians reported to the Department of Justice (DOJ) from 2016 through 2020 reveals that the number of reported hate crime events against Asians increased by 107% in 2020 just in California, USA (Gover et al., 2020). The highest number of anti-Asian hate crime events reported to the DOJ occurred in March and April 2020. This time coincided with President Trump's declaration of a national emergency concerning the COVID-19 pandemic, when the disease continued to cause significant risks to the public health and safety of the Nation (Gover et al., 2020).

Hate crimes are not a new phenomenon, and have existed for centuries, but the spread of racism accelerated dramatically with the advent of social media in recent years. Most social media platforms established strict rules to prohibit criminal and hateful speech but manually controlling all contents posted on the platform to find whether all rules are followed requires copious manual labor. The proposed state-of-the-art solution uses an automatic system to detect such speech and then deliver it to a human content moderator for review and a final decision. This process is the safest and fastest solution. A challenging aspect of detecting hate speech is disagreement on defining hate speech, especially against Asians. Different definitions cause some content to be considered hate speech to some people, not others. Therefore, we need to consider the fixed definition of this matter.

Below, we summarize the most widely used hate speech definitions:

1. Twitter: “Violence against or directly attack or threaten other people based on race, ethnicity, national origin, caste, sexual orientation, gender, gender identity, religious affiliation, age, disability, or severe disease.” (Twitter, 2022)
2. Facebook: “A direct attack against people—rather than concepts or institutions—on the basis of what we call protected characteristics: race, ethnicity, national origin, disability, religious affiliation, caste, sexual orientation, sex, gender identity, and serious disease. We define attacks as violent or dehumanizing speech, harmful stereotypes, statements of inferiority, expressions of contempt, disgust or dismissal, cursing and calls for exclusion or segregation.” (Facebook, 2022)
3. YouTube: “When it incites hatred or violence against groups based on protected attributes such as age, gender, race, caste, religion, sexual orientation, or veteran status.” (YouTube, 2022)
4. Cambridge University: “public speech that expresses hate or encourages violence toward a person or group based on something such as race, religion, sex, or sexual orientation.” (Wikipedia contributors, 2021)
5. Encyclopedia of the American Constitution: “Speech that attacks a person or group based on attributes such as race, religion, ethnic origin, national origin, sex, disability, sexual orientation, or gender identity.” (Levy et al., 2000)
6. Davidson et al.: “Language that is used to express hatred toward a targeted group or is intended to be derogatory, to humiliate, or to insult the members of the group.” (Davidson et al., 2017)
7. de Gilbert et al.: “Hate speech is a deliberate attack directed toward a specific group of people motivated by aspects of the group's identity.” (De Gibert et al., 2018)
8. Fortuna et al. “Language that attacks or diminishes, that incites violence or hate against groups, based on specific characteristics such as physical appearance, religion, descent, national or ethnic origin, sexual orientation, gender identity or other, and it can occur with different linguistic styles, even in subtle forms or when humor is used.” (Fortuna and Nunes, 2018).

In some of the above definitions, hate speech attacks individuals, while in others, it is against groups (MacAvaney et al., 2019). In Parekh (2012) and Fortuna and Nunes (2018), hate speech targets an individual or group based on “an arbitrary or normatively irrelevant feature,” showing the target as an “undesirable presence and a legitimate object of hostility.” Based on previous studies of racial hate from the social and information science literature, we define anti-Asian hate as follows:

- “Online Anti-Asian hate speech is language that contains hate speech toward individuals or groups of Asians. We define online hate speech as profanity, offensive language, or toxicity on social media in individual posts or comments on someone's post. These comments are disrespectful or rude and can result in negative online and offline consequences for Asian individuals, communities, and society.”

As a baseline in this article, we build on the research performed by He et al. (2021). We enhance their dataset by relabeling it four more times by Asian and Asian-American annotators. To perform a study, we have submitted an IRB application that has been accepted by The Human Research Protection Program (HRPP) at City College#2021-0638. We recruited four students as annotators to perform annotation tasks, and the subjects received course credit as compensation for their participation in the research.

Our goal is to improve the He et al. (2021) performance at Asian hate speech detection. Therefore, we used the first part of the labeled tweets used in their study. In the future, we will use more data collected from Twitter for further analysis. Our current research includes the following steps:

1. We used the 2,400 labeled tweets of our baseline and then cleaned data by removing duplicated and non-Asian tweets.
2. We re-annotated the baseline's labeled dataset by four Asian-Annotators.
3. We combined the annotations using majority voting between our annotators' labels and He et al.' labels for each tweet.
4. We measured the agreement rate between our annotators for each tweet and a total agreement for the dataset using Fleiss' Kappa (Fleiss, 1971).
5. We improved the low agreement rate by eliminating tweets that annotators had a lower agreement on. To achieve this, we set a threshold of an acceptable agreement rate by exploring how performance is affected by the amount of data as well as the agreement of annotators on that data.
6. To validate our research, we explored many popular natural language processing feature representation methods and supervised machine learning, and deep learning classification algorithms on He et al. labeled dataset and our improved annotated dataset (Refer to Section 4.2).

The remainder of this article is organized as follows: We explore the literature on hate speech detection in Section 2. Our data organization is explained in Section 3. Various methods of machine learning and our experiment results are presented in Section 4.

## 2. Related studies

The literature on hate speech detection using social media and online content is extensive. A considerable number of Asians have reported increased anxiety and depression due to the racism, verbal, and physical attacks caused by the recent pandemic (Wu et al., 2021).

Several researchers have introduced a new dataset to detect hate toward Asians during COVID-19 (He et al., 2021). In Alshalan et al. (2020), Arshalan et al. identified hate speech regarding the online posts and comments on Twitter during the COVID-19 pandemic posted in Arab countries. In their approach, a convolutional neural network (CNN) model is pre-trained by giving each tweet a score between zero and one, where one is the most hateful text and zero is non-hateful. Also, nonnegative matrix factorization is used to discover the main topics discussed in hate tweets that caused hate speech. Pitsilis et al. (2018) addressed the question “How to effectively identify the class of a new posting, given the identity of the posting user and the history of postings related to that user?”. Their training dataset includes short texts that are labeled by three classes Neutral (N), Racist (R), and Sexist (S). They applied a variant of Recurrent Neural Network called Long-Short Term (LSTM) to achieve their goal. Their study was novel in terms of several extra features concerned with the users' tendency toward hateful behavior and its architecture. Their model combines the output of various LSTM classifiers to improve the classification's performance. They have chosen 16K tweets as their dataset from Waseem and Hovy (2016). Wassem and Hovy developed a model for hate speech detection on Twitter. They manually annotated the dataset to monitor the hate distribution. Later, Waseem (2016) extended their dataset, which was already published, to compare amateur and expert annotations. Finally, they concluded that amateur annotators are more willing to label tweets as hate speech than expert annotators. in Salminen et al. (2020) discussed the problem of lacking models for hate detection on Twitter. A total of 1,97,566 comments from four platforms, Twitter, Reddit, YouTube, and Wikipedia, are collected in their study. They concluded that online hate interpretation varies between individuals; hence, they applied aggregation methods to all classifiers. These methods include majority vote, mean score, and consensus to determine whether a comment could be hateful. Gaydhani et al. (2018) designed a machine learning model which can differentiate between hate speech and offensive language on Twitter by using n-gram and term frequency-inverse document frequency (TFIDF) as features and comparing results obtained using Logistic Regression, Naive Bayes, and Support Vector Machines as classifier models. They used a dataset that includes 16,000 annotated tweets gathered by Waseem (2016). By the given set of tweets, their goal was to classify them into clean, hateful, and offensive classes. They found that Logistic Regression performs better for n-gram and TFIDF features after tuning the hyperparameters by 95.6% accuracy.

Our baseline is the Bing Hu study (He et al., 2021), which focused on hate crimes against Asians during the COVID-19 Pandemic on Twitter. They collected a large dataset of anti-Asian hate and counter hate speech on Twitter related to the COVID-19 pandemic. The annotated 2,000 unique tweets based on the hatefulness toward Asians as hate, counter-hate, or neutral tweet. Then they built a text classifier to identify hate and counter-hate tweets automatically. The similarity of our study with them is that we also made a system to automatically recognize hateful tweets against Asians during COVID-19. Still, our improvement is that we made a more accurate training dataset to get better performance results, which we will explain in the next section.

## 3. Dataset

Twitter has strict rules for publishing tweets and all scientists who are using Twitter API have to follow these rules. Based on these rules, nobody has permission to publish the text of the tweets. Hence, all available annotated tweets include only the ID and label of the tweet without its text. To use these datasets, we need to exploit Twitter API again to retrieve actual texts through the tweets' IDs. This process is challenging because if a tweet was genuinely recognized as racism or hateful, Twitter removed it. The authors' account of these tweets was already suspended most of the time.

On the other hand, the Anti-Asian speech during COVID-19 is a new phenomenon that a few studies have addressed. Therefore, there are not any or enough annotated datasets related to Anti-Asian hate or counter-hate tweets, which were our motivation for building our test and train datasets.

### 3.1. Tweet collection

To tackle the aforementioned gap, we made our large and updated dataset formed by more than 10 million tweets. Each contains all information, including text, ID, location, number of retweets, author's ID, and Geo IDs, which builds our data set to 89 columns. We call our dataset CovAA10M, and according to the Exemption granted to us by IRB (File #2021-0638), we keep this dataset in a secure and password-protected environment.

To collect all tweets related to both “*COVID*−19” and “*Asianhatecrimes*,” the Logical AND operator was used in the building of the Twitter API V.2. We retrieved these tweets in two following phases (Refer to Section 3):

1. We used keywords related to our research from the literature, and we downloaded tweets since March 2020 that COVID-19 got a vital topic for everyone.
2. We extracted all hashtags from downloaded tweets to find new keywords to collect a new dataset.

We hand-labeled a subset of our Comprehensive dataset, and in the current research, we use our hand-labeled dataset to improve the result of one of the previous studies. In our future study, we will create a textual classifier to label the rest of our dataset, and we will use more columns of our dataset to find a network of hate against Asians, but here, we just focus on the text of the tweet. In this section, we explain the dataset creation steps.

To collect our dataset, we have used Twitter API V.2, which Twitter officially released in late August 2020. This new version has improved and can be implemented in either Python IDE such as Jupyter or a new environment called Twarc. The Twarc can be used in the Terminal for IOS and CMD or shell for Windows. Twitter awarded us access to the academic version of this API since this version of the Twitter API is free for Ph.D. students and their advisors. Using this version, we can download up to 10 million tweets per month without restrictions, which means we can access all tweet information.

The previous free version of Twitter API was limited to downloading tweeted tweets from now up to the last 2 weeks to retrieve only 1% of tweets for each keyword. In addition, for each query, we were able to use only one keyword and were limited in time, which means that every 15 min, we had access to only 100 tweets by one query. Hence, using the old version of Twitter API, we were limited in terms of time, information, and the number of tweets.

The new API has flexibility by using the logical operators between keywords. We can retrieve tweets containing many keywords by AND operator and tweets that include only one of the keywords by the OR operator. For example, we made a query to retrieve all tweets that contain both #COVID-19 AND #ChinaLiedPeopleDied keywords from 1 January 2020 to 1 December 2020, and one of the tweets returned to us is the following:

- #china is a threat to the #world. #ChinaVirus #ChinaLiedPeopleDied #Chinazi #ChinaMustPay @ChineseEmbinUS @Chinamission2un #coronavirus #WuhanVirus #COVID19 #HongKong #Europe #Australia https://t.co/IhJo19wk1P.

We have downloaded around 10 million tweets related to both COVID-19 and Anti-Asian. To achieve this goal, we have taken the following preparations:

1. We have downloaded only tweets that were tweeted during the COVID-19 pandemic from 02/01/2020 to 07/14/2021.
2. Our initial keywords are hashtags that were made during the COVID-19 pandemic for the hate or counter hate of Asians, such as #ChinaLiedAndPeopleDied, #ChinaVirus, #Chinese virus, and #ChineseBioterrorism.
3. To extend the range of keywords to download new tweets, we extracted all hashtags from downloaded tweets, and then we recognized new hashtags as our new keywords.

### 3.2. Annotation

In some previous studies, a tweet was labeled hateful if any hateful keywords or hashtags were included. Conversely, the tweet was labeled as counter-hate if it consisted of any prominent hashtag for supporting the targeted group. This method is not trustworthy since even a tweet may include the hateful hashtag(s), but simply categorizing it based on the presence or absence of recognized hateful hashtags is not sufficient and accurate classifying. The reason is that hashtags are often added to the text of the tweets to achieve more visibility. A tweet can be hateful without having an abusive hashtag or even without any hashtag, and this reason is valid for counter-hate tweets. Therefore, we developed an accurate annotating process to establish the ground truth categories of tweets based on the tweet content.

For the annotation task, we have used our online Anti-Asian hate speech definition mentioned in the Introduction Section 1. To stem away from false positives, such as: “I hate owing people favors,” the specific hate targets (e.g., Black people, Mexican people, Chinese, stupid people) have been used, as well as 1,078 hate words from the “Hatebase.org,” which is a database of hateful words and phrases (Salminen et al., 2020).

Based on our online Anti-Asian hate speech definition, we define three classes that are also COVID-related as follows:

#### 3.2.1. Hateful

The hateful Anti-Asian COVID-19 speech is an antagonistic speech that:

1. has one or more COVID-19 keywords,
2. is directed toward an individual or a group of Asian people or their organizations, countries, governments,
3. is abusive, disparaging, or blaming for the making, spreading, misrepresentation, or mismanagement of COVID-19.
  - Example: "It's the Chinese virus, from China, caused by your disgusting eating habits, your cruelty. Boycott anything Chinese #kungflu #chinaliedpeopledied #covid (He et al., 2021)"

#### 3.2.2. Counterhate

The counterhate Anti-Asian COVID-19 speech is a supportive speech that:

1. recognizes, criticizes, and actively opposes hate speech that motivates racism or violence toward Asian people or their communities, countries, or governments because of the COVID-19 pandemic. This kind of speech is the direct reply to hateful tweets.
2. support and defend the Asian people, community, country, or government. (stand-alone tweets)
  - Example: "The virus did inherently come from China but you cant just call it the Chinese virus because thats racist. or KungFlu because 1. Its not a f*****g flu it is a Coronavirus which is a type of virus. And 2. That's also racist." (He et al., 2021)

#### 3.2.3. Neutral

The neutral Anti-Asian COVID-19 is a speech that:

1. Neither explicitly nor implicitly hateful nor counterhateful about Asians but still contain content related to COVID-19 and Asians.
2. mostly are news-related, advertisements, or outright spam
  - Example: "COVID-19: #WhiteHouse Asks Congress For $2.5Bn To Fight #Coronavirus: Reports #worldpowers #climatesecurity#disobedientdss #senate #politics #news #unsc #breaking #breakingnews #wuhan #wuhanvirus https://t.co/XipNDc” (He et al., 2021)

## 4. Proposed approach

The first step in our proposed method is preparing the accurate annotated dataset. Because our baseline for this research is He et al.'s study, hence for the annotation task, we used their annotated dataset. This dataset is available on their project website: “http://claws.cc.gatech.edu/covid”.

The size of this dataset is 2,400 tweets. We removed all duplicated and non-Asian-related tweets, which resulted in 1,901 tweets. Furthermore, we removed all identifiers from them based on our IRB approval. Afterward, we distributed these tweets between four annotators within separate files. We uploaded each file to a Google spreadsheet by adding the definitions of three classes and 17 labeled tweets as examples to each spreadsheet. Each spreadsheet has three columns: 1) the text of the tweet, 2) the label of that tweet, and 3) a comment explaining why that label is chosen. We shared each spreadsheet with an Asian student for the labeling task. We used Asian Annotators because they are more familiar with our topic and better understand the meaning of Anti-Asian rather than the other students. Students must choose a number between one to five as a label of the tweet, where one means Extremely Counter-hate, two means Counterhate, 3 means Neutral, 4 means Hateful, and five is Extremely Hateful. Each file is repeatedly annotated by all four students, which means each tweet has four labels which are numbers. The difference between each two assigned numbers has been calculated for each tweet. If the result was greater equal 3, then its tweet has been discussed among students to resolve disagreement and update the labels. Finally, assigned numbers were converted to one of the categorical classes where Counterhate replaces 1 and 2, 3 is replaced by Neutral, and 4 and 5 are replaced by hate. We first used five numbers as labels and then replaced them with three classes to make a more accurate training dataset in which annotators have the highest agreement for each of the three labels. Afterward, we applied the most famous machine learning and deep learning methods to find the system's performance. We tried to improve the performance of the system by tuning our dataset. In Sections 4.1 and 4.2, we will explain our approach in detail.

### 4.1. Pre-processing

In natural language processing approaches, we need to perform some pre-processing steps on the dataset before using any classification or prediction algorithm. Pre-processing is the process of removing noise and elements that do not matter in sentiment analysis or opinion mining tasks. For our study, this process includes lowercasing, tokenizing, and removing punctuation, user mention sign (@), hashtags(#), duplicate characters, URLs, stopwords, and images.

### 4.2. Methods

Many types of research have been done to identify hateful content on online platforms and social media. The fundamental method in hate speech detection is a lexicon-based approach, representing based on the hateful keywords. Exploiting external sources such as the HateBase lexicon leads to a high-performing system in hate speech detection but maintaining and upgrading these resources are challenging (MacAvaney et al., 2019). Furthermore, using specific hateful keywords in training data results in many false negatives related to the hateful samples, which are not containing those keywords (Davidson et al., 2017; MacAvaney et al., 2019). Hence, we do not employ such external resources in this study; instead, we have exploited the most famous methods in machine learning and deep learning methods.

#### 4.2.1. Machine learning approach

A variety of machine learning approaches have been employed in the literature (Waseem, 2016) to detect hateful and abusive content. It is claimed that the most predictive features in this task are surface-level features such as a bag of words, word-level, and character-level n-grams. By reviewing the literature, we have decided to employ all famous machine learning methods in the literature, which is already proven that they have a good performance in sentiment analysis, opinion mining, and speech detection. These methods are briefly explained in the following:

**Naïve Bayes:** Jurafsky and Manning (2009) is a probabilistic classifier that builds statistical models of classes from the training dataset. It is called naive because it is a Bayesian classifier that simplifies assumptions or naïve about how features interact. This probabilistic classifier, given document d, among all classes c ∈ C returns class c with the maximum posterior probability. The Bayes theorem is represented as follows:


(1) $$\begin{array}{l} P\left(\theta |x\right)=\frac{P\left(x|\theta \right).P\left(\theta \right)}{P\left(x\right)} \end{array}$$

where the *P*(θ|**x**) is the posterior probability, *P*(*x*) is the normalization constant, *P*(**x**|θ) is the likelihood, and *P*(θ) is the prior probability for the parameters we know before experimenting.

The class label for a test document d = {d_1_, d_2_ ,.., d_n_}, where d_j_ is the frequency of feature j in document d, is predicted using the Bayes rule as follows:


(2) $$\begin{array}{l} label\left(d\right)=arg_{\text{c}}maxP\left(c|d\right) \end{array}$$

We know $P\left(\text{c}|\text{d}\right)=\frac{P\left(\text{d}|\text{c}\right).P\left(\text{c}\right)}{P\left(\text{d}\right)}$. Hence:

$label\left(d\right)=arg_{\text{c}}max\frac{P\left(\text{d}|\text{c}\right).P\left(\text{c}\right)}{P\left(\text{d}\right)}=$

$arg_{\text{c}}max\left(\frac{P\left(\text{d}|\text{c}{}_{1}\right).P\left(\text{c}{}_{1}\right)}{P\left(\text{d}\right)},\frac{P\left(\text{d}|\text{c}{}_{2}\right).P\left(\text{c}{}_{2}\right)}{P\left(\text{d}\right)},...,\frac{P\left(\text{d}|\text{c}{}_{\text{n}}\right).P\left(\text{c}{}_{\text{n}}\right)}{P\left(\text{d}\right)}\right)$

Because we are always asking about the most likely class for the same document d that has some probability P(d), therefore, the denominator P(d) could be dropped:


(3) $$\begin{array}{l} label\left(d\right)=arg_{\text{c }\in \text{ C}}maxP\left(d|c\right)P\left(c\right) \end{array}$$

Document d could be represented as a set of features f_1_, f_2_, …, f_n_, therefore:


(4) $$\begin{array}{l} label\left(d\right)=arg_{\text{c }\in \text{ C}}maxP\left(f_{1},f_{2},\ldots ,f_{\text{n}}|c\right)P\left(c\right) \end{array}$$

**Logistic Regression:** Naïve Bayes is a generative model. This model assigns a class c to a document d by computing a likelihood and a prior and not just by directly computing *P*(*c*|*d*). This process expresses how to generate the features of a document if we knew it was class c. Dissimilarly, Logistic Regression is a discriminative model that attempts to compute *P*(*c*|*d*) directly. It learns to assign a high weight to each document feature that immediately improves its ability to discriminate between possible classes. This action may not generate an example of one of the classes (Jurafsky and Manning, 2009).

**Support Vector Machine (SVM):** SVM is a popular classifier for text classification and is often considered the state-of-the-art classifier. SVM classification is ideal for text data because of the sparse nature of the text, in which few features are irrelevant. Still, they tend to be correlated and generally classified as linearly separable categories (Joachims, 1997). SVM can build a nonlinear decision surface in the original feature space by mapping the data instances non-linearly to an inner product space. Furthermore, the classes can be separated linearly with a hyperplane (Aizerman, 1964).

**K-nearest neighbors (KNN):** Fix and Hodges (1952) KNN is a simple and commonly used non-parametric and instance-based learning algorithm that is used for both classification and regression tasks. Non-parametric means that the algorithm is without any knowledge and does not make any assumptions related to the underlying data distribution. Conversely, the instance-based means that the algorithm does not use the training instances to generalize during the training phase. The training phase in KNN is high-speed, and the training instances are used in the testing phase to classify an unseen example. The advantage of KNN is that it is a simple algorithm with no assumptions about the data and could be applied to classification and regression tasks. The drawback of this algorithm is that it is costly in the use of memory since there is a need to store all training data. Also, it is costly in time because it needs to compare each new instance to all the training instances and calculate the similarity.

**Decision Tree:** This technique is a map of the possible outcomes of a series for the related choices that uses a tree-like structure graph. The three types of nodes that establish the decision tree are (i) decision, (ii) chance, and (iii) end nodes. The decision tree can be linearized into decision rules, but this may conclude in deep paths. This algorithm is tested by Bourgonje et al. (2017), achieving a 76.17 f-score when tested in English Tweets.

**Random Forest:** This method is an ensemble learning method for tasks that grow many classification trees such as classification and regression. To classify a new object from an input vector, it feeds the input vector down each tree in the forest. Each tree gives a classification output by labeling the input or a ranking result by sorting the most possible labels. The forest chooses the classification having the most votes (over all the trees in the forest). To avoid overfitting, the bootstrap aggregating technique is used, which is a machine learning ensemble meta-algorithm designed to improve the stability and accuracy of machine learning algorithms used in statistical classification and regression. This method is used in Gaydhani et al. (2018) and Vidgen and Derczynski (2020) with no significant results, since in both studies, the model that outperformed all others was Logistic Regression.

**Extreme Gradient Boosting (XGBoost):** XXGBoost applies to boost, which trains each new instance to emphasize the previous training instances modeled for better classification results. It is a combination of classification and regression trees (CART) (Breiman et al., 2017) but re-defined the objective function with both training loss and complexity of the trees to decrease the chance of overfitting. Thus, XGBoost is a powerful model with high extensibility.

#### 4.2.2. Deep learning approaches

Most of the current machine learning learns the predictive value by calling the weights of human-given features. Representation learning systems attempt to learn good representations or features automatically. Deep learning algorithms try to learn multiple levels of representation of increasing complexity or abstraction, and a system with such a sequence is a deep architecture. The vast majority of such study has explored deep belief networks (DBNs) and Markov Random Fields with multiple layers. Several other variants of deep learning have seen a revival due to improved optimization methods of multiple-layer neural networks. In this article, we have explored the following variants of deep learning:

**Long Short-Term Memory (LSTM)** is a special kind of Recurrent Neural Networks (RNNs) that works much better. In theory, RNN can learn long-term dependency, but in practice, they fail it, such as predicting the last word in "I grew up in France…. I speak fluent French." that LSTM solved this problem. LSTMs are explicitly designed to avoid long-term dependency problems and, therefore, are well-suited to classifying, processing, and making predictions based on time series data. All recurrent neural networks have the form of a chain of repeating modules of neural networks, such as a single tanh layer. LSTMs, instead of having a single neural network layer, have four. A standard LSTM unit comprises a cell, an input gate, an output gate, and a forget gate. The cell remembers values over arbitrary time intervals, and the three gates regulate the flow of information into and out of the cell (Hochreiter and Schmidhuber, 1997).

**Bidirectional LSTM (biLSTM)** is a sequence processing model that consists of two LSTMs: one taking the input in a forward direction and the other in a backward direction. BiLSTMs effectively increase the amount of information available to the network, improving the context of the algorithm (e.g., knowing what words immediately follow and precede a word in a sentence). Therefore, as the first improvement of standard LSTM, we use a Bi-Directional LSTM. As the name suggests, this Layer reads text forwards and backward and simultaneously allows the model to get information from past and future states. It also usually provides a nice boost in performance over the single-pass LSTM (Liu et al., 2016).

**Dropout** is also added to reduce overfitting as the number of parameters in the model increases. A Dropout Layer drops data from the input but only during training. This encourages the model to be more robust and not overly dependent on specific signals from the training data to make predictions. Since we have a relatively small data set compared to our model size, dropout is critical to ensure the model doesn't quickly overfit the training set (Deshmukh and Kiwelekar, 2020).

**Convolution:** A modern architecture for Text Classification usually includes the addition of Convolutions stacked on top of each state vector in the LSTM instead of just predicting based on the last state. This technique is borrowed from Image Classification and assumes that related information is locally grouped (a kernel). Taking the average or Max of the Convolutional Layer is common, which is not a sure-fire choice but can sometimes perform better on specific tasks. Depending on the run, this will serve as well or worse than the vanilla Bi-Directional LSTM, though we will apply it in our problem as well (Collins and Duffy, 2001).

**Bidirectional encoding representations from transform:** Devlin et al. (2018) is a new approach published in late 2019 by the researchers at the AI Google language. They designed BERT to pre-train deep bidirectional representations from an unlabeled text by jointly conditioning on both left and right context in all layers. As a result, the pre-trained BERT model can be finetuned with just one additional output layer to create state-of-the-art models for a wide range of tasks, such as question answering and language inference, without substantial task-specific architecture modifications.

In our method, each token(word) converts to a vector. Hence, the encoder makes the vector of vectors as the input of the deep learning algorithms.

**Figure 1:**
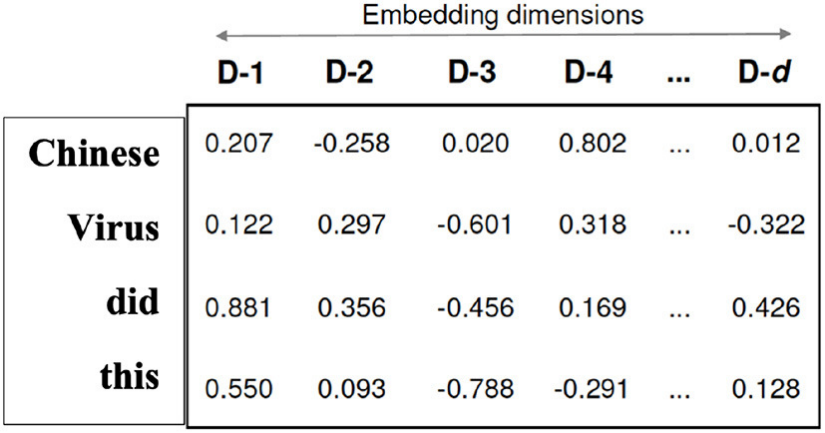
Example of word embedding.

Figure 1 shows the word embedding (word2vec) for a short tweet that includes four words and n-dimensions. The number of dimensions depends on the method we use. For example, BERT uses the Google huge dataset that is pre-trained on a large corpus of unlabeled text, including the entire Wikipedia (that is 2,500 million words) and Book Corpus (800 million words). Each word is embedded in a vector of 300 dimensions.

In **Table 3**, we presented our results for deep learning algorithms. To find the best epoch in the LSTM and BERT model, we performed two experiences:

1. We set the number of epochs at 25; we wrote a function with a loop that updates the best epoch based on the higher f1-score and less validation loss in each iteration.
2. When the number of epochs used to train a neural network model is more than necessary, the training model learns patterns specific to sample data to a great extent which causes overfitting. To avoid this issue, Keras supports the early stopping of training *via* a callback called EarlyStopping. This callback allows us to specify the performance measure to monitor, and the trigger, and once triggered, it will stop the training process. The Early Stopping call back function can monitor either loss or accuracy values. Training comes to a halt either when the loss is being monitored, an increment is observed in loss values, or a decrement is observed in accuracy values when accuracy is being monitored.

## 5. Experimental setup

To validate our research, we have chosen He et al.'s labeled dataset (He et al., 2021) as our baseline, and then we improved the performance of the system by both of the following techniques:

1. Relabeling dataset (Refer to Section 3.2).
2. Enhancing the Fleiss Kappa rate (Explained in Phase 2 Section 5.4 and Phase 3 Section 5.5).

Our study includes three phases: 1) We analyzed the He et al. dataset and then applied all famous classification methods to find the most accurate method for hate detection on this dataset (Section 5.3); 2) We undertook to improve the accuracy of methods by tuning the dataset. We also found the Fleiss Kappa number, and then in the subsequent phases, we tried to enhance this number in order of creating an annotated dataset with a high agreement rate between annotators. We proved that enhancing the Fleiss Kappa resulted in improving the performance (Refer to Section 5.4 and 5.5).

In Section 5.1, we briefly review the Fleiss Kappa measure and its application. In Section 5.2, a different variety of measures are explained.

### 5.1. Fleiss kappa

Cohen's kappa is a measure of the agreement between two raters, where agreement due to chance is factored out. If the number of raters is more than two, we have to do one of the following:

- Calculate Cohen's kappa between each pair of raters for each tweet and then calculate the average of those kappa values to find inter rater reliability.
- Use an extension of Cohen Kappa called Fleiss' kappa, introduced in Fleiss (1971), and calculate a single kappa for all the raters for all possible combinations. To calculate this number, first, we calculated the proportion of agreement for each tweet by the Formula 5:

(5) $$\begin{array}{l} P_{\text{i}}=\frac{1}{n\left(n-1\right)}\overset{k}{\underset{j=1}{\sum }}n_{\text{ij}}\left(n_{\text{ij}}-1\right) \end{array}$$

where n is the number of raters for the i*th* tweet and j*th* class.

Then the rate of agreement for each category is calculated by Formula 6:


(6) $$\begin{array}{l} \frac{\left(TotalAgreement\right)}{\left(NumberOfTweets\right)\times \left(NumberOfRaters\right)} \end{array}$$

Formula 7 represents the overall extent of agreement:


(7) $$\begin{array}{l} \bar{P}=\frac{1}{N}\overset{N}{\underset{i=1}{\sum }}P_{\text{i}} \end{array}$$

Formula 8 calculates the probability if raters made their agreement purely at random:


(8) $$\begin{array}{l} {\bar{P}}_{\text{e}}=\overset{k}{\underset{j=1}{\sum }}P_{\text{j}}^{2} \end{array}$$

Finally, we calculate Fleiss' Kappa by the following formula:


(9) $$\begin{array}{l} k=\frac{\bar{P}-{\bar{P}}_{\text{e}}}{1-{\bar{P}}_{\text{e}}} \end{array}$$

From the literature, although there is no formal way to interpret Fleiss' Kappa's value, the following values show how to interpret Cohen's Kappa's value, which is used to assess the level of inter-rater agreement between just two raters:

- 0 < 0.20 | Poor
- 0.21–0.40 | Fair
- 0.41–0.60 | Moderate
- 0.61–0.80 | Good
- 0.81–1 | Very Good

Therefore, we used these ranges as a Fliess' Kappa rate benchmark to measure the agreement level of annotators in our labeled dataset.

### 5.2. Performance metrics

To evaluate the performance of machine learning and deep learning algorithm in our different phases, we have used a variety of metrics that we briefly defined in the following (Botchkarev, 2018):

**Accuracy** specifies what proportion of tweets were correctly classified for each class.

**Precision** tells us what proportion of tweets that were predicted as hateful is genuinely hateful.

**Recall** shows what proportion of actual hateful tweets in the dataset is correctly classified.

**F1-Score** combines both Precision and Recall for all classes. Unlike accuracy, it does a better job of accounting for any imbalances in the distribution of texts among classes.

**AUC - ROC curve** is a performance measurement for the classification problems at various threshold settings. ROC is a probability curve, and AUC represents the degree or measure of separability, and it tells how much the model is capable of distinguishing between classes.

**Support:** number of samples of the actual response that lie in that class, which here is the Hate class.

**Validation loss** is the loss calculated on the validation set when the data is split to train, validate, and test sets using cross-validation. To tackle the problem of working with imbalanced datasets, we put more data from the larger class of dataset, which here is the Neutral class, in the validation set.

**MAE:** The mean absolute error represents the average of the absolute difference between the actual and predicted values in the dataset. It measures the average of the residuals in the dataset.


(10) $$\begin{array}{l} MAE=\frac{1}{N}\overset{N}{\underset{i=1}{\sum }}|y_{\text{i}}-ȳ| \end{array}$$

where ȳ is the mean value of y

**MSE:** Mean Squared Error represents the average squared difference between the original and predicted values in the data set. It measures the variance of the residuals.


(11) $$\begin{array}{l} MSE=\frac{1}{N}\overset{N}{\underset{i=1}{\sum }}{\left(y_{\text{i}}-ȳ\right)}^{2} \end{array}$$

**RMSE:** Root Mean Squared Error is the square root of Mean Squared error. It measures the standard deviation of residuals.


(12) $$\begin{array}{l} RMSE=\sqrt{MSE}=\sqrt{\frac{1}{N}\overset{N}{\underset{i=1}{\sum }}{\left(y_{\text{i}}-ȳ\right)}^{2}} \end{array}$$

In the following sections, we review each phase in detail.

### 5.3. Phase 1: Defining baseline

**Figure 2:**
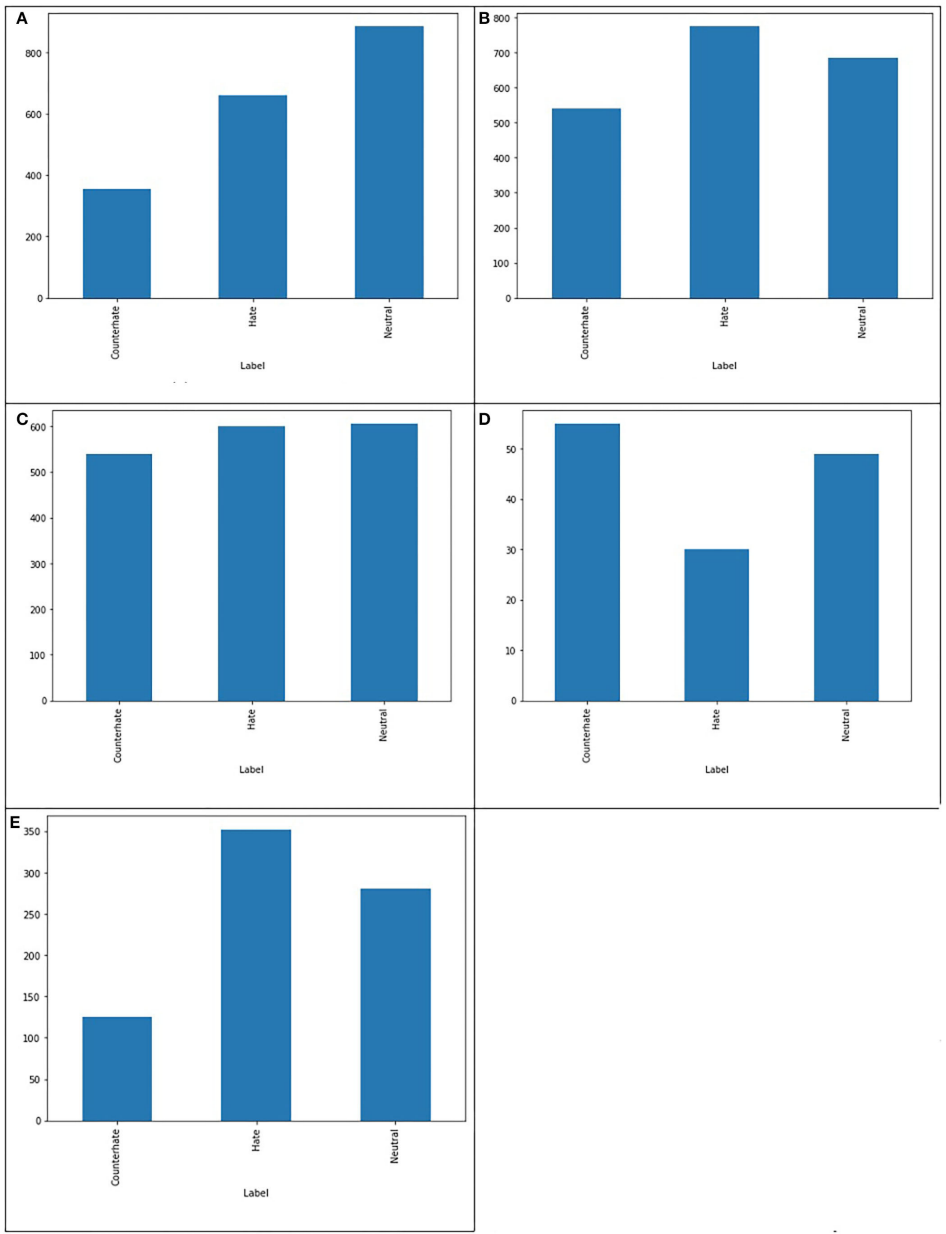
Comparing the balance in the number of tweets in each class in different datasets. **(A)** Baseline dataset, **(B)** Our dataset, **(C)** Our improved dataset, **(D)** Ambiguous dataset, and **(E)** Dataset with full agreement.

**Figure 3:**
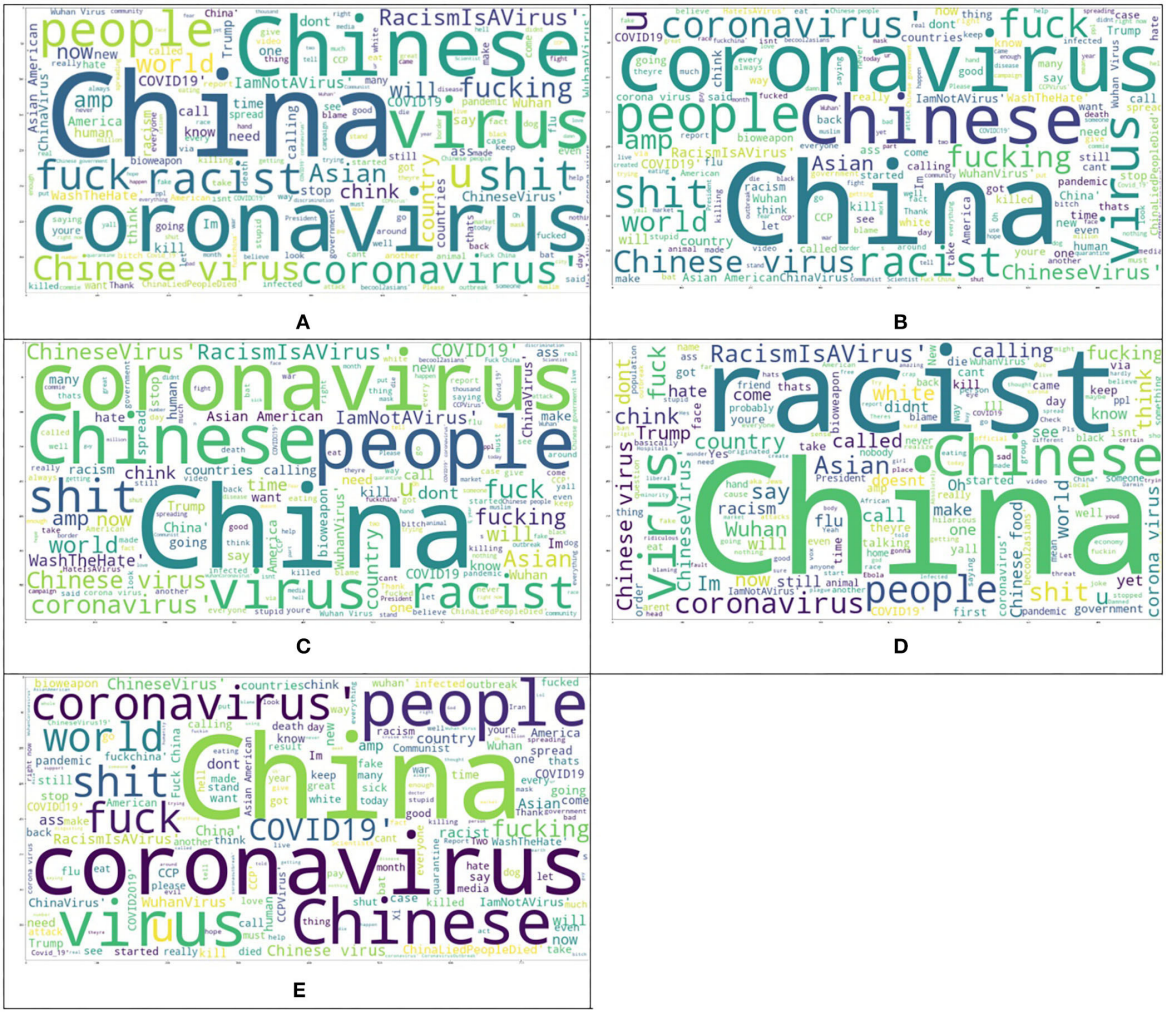
Comparing the wordcloud of different datasets. **(A)** Baseline dataset, **(B)** Our dataset, **(C)** Our improved dataset, **(D)** Ambiguous dataset, and **(E)** Dataset with full agreement.

After removing duplicate and Non-Asian tweets from our baseline dataset (He et al., 2021), the amount of 1,901 unique tweets is left that each tweet belongs to one of the three classes of hate, neutral and counter-hate. The distribution of classes in this dataset is shown in Figure 2A. From this figure, it is easily visible that this dataset is imbalanced. In the subsequent phases, we aim to balance the dataset and improve the system's performance in a way that we lose data as little as possible. The Fleiss' Kappa's number we achieved for the HE et al. labeled dataset by the method from the previous section is 0.36, interpreted as Fair. We will enhance this number in the following phases to investigate how this number can affect the system's performance. The most frequent words in this dataset are demonstrated in Figure 3A as the Wordcloud chart. In this visualization, it is noticeable that China, Chinese, and Coronavirus are the dominant words in all tweets of this dataset. To understand how people from each tendency talked about Asians during COVID-19, we also found the words and phrases correlated to each class by finding the most frequent unigrams, bigrams, and trigrams for each category in each dataset. The most frequent terms in He et al.' dataset are shown in Table 1a, which indicates haters had a discriminatory view of the Chinese by calling the coronavirus the Chinese virus, and conversely, supporters demanded to stop calling coronavirus as Chinese. We divided our experiment into two main categories: machine learning methods and deep learning methods. Then, we applied all famous algorithms from the literature that we defined earlier to find the most accurate one on this dataset. The result of this research for this phase is shown in Tables 2a, 3a. This result shows that the logistic regression achieved the best performance of accuracy of 0.75 in machine learning methods, and the BERT algorithm, with an F1-score of 0.71, is the best in deep learning methods.

**Table 1:** Most frequent phrases for each category in each dataset.

		**Classes**	**Unigram**	**Bigram**	**Trigram**
(a)	Base line dataset	Counterhate	Stand	Calling Chinese	Stop Calling Chinese
			Asian	Asian friends	Calling Chinese virus
		Neutral	Chinese	Chinese virus	Fuck Chinese virus
			Coronavirus	Fuck China	Fucking Chinese virus
		Hate	Chinaliedpeopledied	Fuck Chinese	Fucking Chinese virus
			Fuck	Fuck China	Fuck Chinese virus
(b)	Our dataset	Counterhate	Iamnotavirus	Asian American	Hate Asian Americans
			Asian	Asian Americans	Calling Chinese virus
		Neutral	Cases	Fuck China	Diamond Princess cruise
			Coronavirus	Cruise ship	COVID-19 Coronavirus wuflu
		Hate	Fuck	Fuck chinese	Fucking Chinese virus
			Chinaliedpeopledied	Fuck China	Fuck Chinese virus
(c)	Our improved dataset	Counterhate	Racismisavirus	Asian American	Hate Asian Americans
			Asian	Asian Americans	Calling Chinese virus
		Neutral	Bioweapon	Chinese virus	Diamond princess cruise
			Coronavirus	Fuck China	COVID-19 coronavirus wuflu
		Hate	Fuck	Fuck Chinese	Fuck Chinese virus
			Chinaliedpeopledied	Fuck China	Fucking Chinese virus
(d)	Ambiguous dataset	Counterhate	World	Chinese virus	**——–**
			Chink	Corona virus	
		Neutral	Coronavirus	Chinese virus	**——–**
			Fuck	Corona virus	
		Hate	Fuck	Chinese food	**——–**
			Started	Corona virus	
(e)	Dataset with full agreement	Counterhate	Asian	Asian people	Fuck Chinese virus
			Racismisavirus	Calling Chinese	Calling Chinese virus
		Neutral	Chinese	Fuck China	Calling Chinese virus
			Coronavirus	Chinese virus	Diamond princess cruise
		Hate	Racismisavirus	Fuck Chinese	Fuck China fuck
			Coronavirus	Fuck China	Fuck Chinese virus

**Table 2:** The performance of machine learning methods for different phases.

		**Model**	**Accuracy**	**F1_score**	**Precision**	**ROC_AUC**	**MAE**	**MSE**	**RMSE**
(a)	Phase 1	R.F	0.73	0.68	0.74	0.72	0.27	0.27	**0.52**
		K.N.N	0.65	0.64	0.61	0.65	0.36	**0.4**	0.6
		S.V.M	0.73	0.7	0.72	0.73	0.27	0.27	**0.52**
		XGBoost	0.68	0.65	0.66	0.68	0.32	0.32	0.57
		**L.R**	**0.75**	**0.72**	**0.73**	**0.75**	0.25	0.25	**0.52**
		D.T	0.66	0.65	0.63	0.66	0.25	0.25	0.5
		N.B	0.64	0.52	0.66	0.62	**0.36**	**0.4**	0.6
(b)	Phase 2	R.F	0.75	0.49	0.8	0.65	0.25	0.25	0.5
		K.N.N	0.72	0.54	0.62	0.67	**0.41**	**0.4**	**0.64**
		S.V.M	0.8	0.65	**0.82**	0.74	0.19	0.19	0.44
		XGBoost	0.78	0.6	0.8	0.71	0.21	0.21	0.46
		**L.R**	**0.8**	**0.69**	0.72	**0.76**	0.2	0.2	0.44
		D.T	0.71	0.58	0.57	0.68	0.2	0.2	0.45
		N.B	0.59	0.5	0.42	0.6	**0.41**	**0.4**	**0.64**
(c)	Phase 3	R.F	**0.8**	0.62	0.74	0.72	0.2	0.2	0.45
		K.N.N	0.73	0.48	0.59	0.64	**0.38**	**0.4**	0.62
		S.V.M	0.78	0.56	0.75	0.7	0.22	0.22	0.47
		XGBoost	0.77	0.55	0.68	0.69	0.23	0.23	**0.48**
		**L.R**	**0.8**	**0.63**	**0.73**	**0.73**	0.2	0.2	0.4
		D.T	0.77	0.63	0.61	0.73	0.2	0.2	0.45
		N.B	0.62	0.52	0.42	0.63	**0.38**	**0.4**	0.62

*From the literature, we know that F1-score and ROC-AUC have been designed to work well on imbalanced data, and also F1-score combines precision and recall metrics; hence, we simply can conclude that Logistic Regression has a better overall performance in all phases. The best result is bolded in each column, meaning the smallest number for MAE, MSE, and RMSE, and the largest number for the rest is our favorite. In each phase, a method with the overall best performance is highlighted in yellow*.

**Table 3:** The performance of deep learning methods for different phases.

		**Model**	**Best epoch**	**Validation loss**	**Precision**	**Recall**	**support**	**F1-score**	**Test accuracy**	**MAE**	**MSE**	**RMSE**
(a)	Phase 1	Basic LSTM	6	0.97	0.55	0.73	63	0.63	0.61	0.31	0.18	0.43
		Bidirectional LSTM	6	1.79	0.67	0.73	63	0.7	0.65	0.23	0.2	0.45
		Bi. LSTM with dropout	6	2.6	0.66	0.6	63	0.63	**0.6**	0.26	0.23	0.48
		Convolution	6	1.61	0.66	0.67	63	0.66	0.66	0.23	0.19	0.43
		**BERT**	**4**	**1.59**	-	-	-	**0.71**	-	-	-	-
(b)	Phase 2	Basic LSTM	7	0.91	0.62	0.79	68	0.7	0.65	0.29	0.17	0.41
		Bidirectional LSTM	7	1.69	0.71	0.71	68	0.71	0.66	0.22	0.19	0.44
		Bi. LSTM with dropout	7	1.59	0.64	0.81	68	0.71	0.66	0.22	0.19	0.44
		Convolution	7	1.56	0.62	0.81	68	0.7	0.66	0.23	0.19	0.43
		**BERT**	**1**	0.75	-	-	-	**0.74**	-	-	-	-
(c)	Phase 3	Basic LSTM	6	0.84	0.71	0.89	62	0.79	0.61	0.3	0.16	0.4
		Bidirectional LSTM	6	0.65	0.74	0.84	62	0.79	0.72	0.19	0.17	0.41
		Bi. LSTM with dropout	6	0.63	0.76	0.76	62	0.76	0.71	0.2	0.16	0.41
		Convolution	6	0.58	0.71	0.76	62	0.73	0.69	0.21	0.17	0.41
		**BERT**	**3**	0.64	-	-	-	**0.85**	-	-	-	-

*The favorite is the higher F1-score and fewer epochs and validation loss. Hence, BERT has better performance in all three phases, and we achieved higher performance with fewer epochs in phase three, which outperformed our baseline performance. In each phase, a method with the overall best performance is highlighted in yellow*.

### 5.4. Phase 2: Making our annotated dataset

As we already mentioned, we annotated 3,000 tweets from our CovAA10M dataset by four Asian students. These tweets and informed consent are given to participants in writing and form of the Excel sheets. Participants are Asian students who have good skills in reading and writing English. For this phase of our research, we used 1901 of them which are the same as the He et al. training dataset. We labeled each tweet four times by counting He et al. labels, each tweet is labeled five times. Furthermore, we found the agreement of each tweet between five annotators and then we calculated the Fleiss Kappa rate for the entire dataset. In this phase, we have to use Fleiss Kappa, not Cohen's Kappa, because more raters are than two. The Fleiss Kappa for our dataset consisting of 1,901 tweets and five labels for each, is 0.53 which is interpreted as moderate, which means we enhanced it from the baseline. Figure 2B shows the distribution of tweets for the three classes in this phase. By comparing Figures 2A,B, it is easily recognizable that our dataset is more balanced rather than the baseline's dataset. Figure 3B visualize the wordcloud of our annotated dataset, which shows still words Chinese and Coronavirus are the most common words. Table 1b, represents what people from each category talk about Asians during COVID-19. Based on this result, haters are insisting that Coronavirus is Chinese and that China is responsible for the death of innocent people by saying lies. On the other hand, supporters made a slogan of “I am not a Virus” to express nobody can not treat Chinese people like a virus. For classification purposes, we add another column to our dataset, including majority voting for each tweet of the dataset. The performance for different machine learning classifiers is shown in Table 2b, which indicates again logistic regression was the winner, but this time the accuracy is 0.05 higher than in the previous phase. In the deep learning section, which the performance of algorithms is shown in Table 3b, again, the BERT algorithm is the winner by an F1-score of 0.74.

### 5.5. Phase 3: Improving phase 2's dataset

In this phase, we aimed to improve the Fleiss Kappa by Finding a threshold to eliminate tweets with a lower agreement between their annotators. Our labeled dataset from phase 2 consists of 1901 rows, and each row includes the text of the tweet, five labels, and the majority voting of the five raters. The distribution of tweets for three classes in this dataset is the following: Counter-hate: 507 tweets; Hate: 742 tweets, Neutral: 652 tweets.

To balance data and improve Fleiss Kappa, we eliminate tweets with low agreement from Neutral and Hate classes with the following agreement thresholds:

- Hate: ”*agreement”* < 0.6. which causes to eliminate 122 tweets.
- Neutral: ”*agreement”* < 0.4. which causes to eliminate 16 tweets.

Hence, the statistic of our new dataset is 1,829 tweets in total with the following distribution: Counterhate: 507; Hate: 653; Neutral: 669.

After the above process, the Fleiss' Kappa number from 0.5297 enhanced to 0.591356 which still is Moderate. We need a little more improvement to fall in the good category of Fleiss Kappa number. We perform it by eliminating a few more Hateful/Neutral tweets. Previously, we have removed all hateful tweets with an agreement of less than 0.6 and all Neutral tweets with an agreement of less than 0.4. There are 267 tweets with an agreement equal to 0.6 and if we remove all of them, then we will miss a lot of data, and also, we will make our dataset imbalanced because of lacking hateful tweets. Hence, we randomly eliminated part of the Neutral and hateful tweets to enhance Fleiss'Kappa and balance our data. There are 75 neutral tweets with an agreement rate equal to 0.4. Because of the higher probability that the semantic of the tweet falls in the neutral category, we have decided to eliminate more tweets from the Neutral class rather than the hate class. Hence, we randomly removed 20 tweets whose agreement rate is equal to 0.4, and 10 tweets from the Hate class whose agreement rate is equal to 0.6. A new distribution of our dataset is as the following: Counter-hate: 507; Hate: 580; Neutral: 596.

Therefore, the new Fleiss'Kappa number is enhanced from 0.591356 to 0.614478 and categorized as Good.

To improve Fleiss Kappa from 0.5297 to 0.614478, we only missed 11.46% of data from 1,901 to 1,683 tweets. Figure 2C shows the distribution of our improved dataset, which clearly can be observed that our dataset is almost becoming balanced. This is while we have balanced our data set in such a way that we just missed a very negligible amount of data that five annotators had the lowest amount of agreement of them; hence, we can consider them as noise. The Wordcloud visualization shown in Figure 3C emphasizes that China is still the center of attention. Table 1c, shows the analysis of people's thoughts from different groups. This analysis indicates that although haters continue insulting the Chinese and making them responsible, supporters have gone a step further and recognized this hate as racism. We carried out the same result as the previous phase in the classification task with the machine learning algorithms, but we achieved our highest performance in the deep learning part by the BERT algorithm with the F1-score of 0.85.

## 6. Conclusion and discussion

We presented a study on recognizing hate speech toward Asians on the Twitter platform during COVID-19. Our contributions in this study are 1) We created a dataset consisting of 10 million tweets related to both COVID-19 and Asians. 2) We chose a baseline that is recent research similar to our topic. 3) We annotated 3,000 tweets by four Asian annotators, and 1,901 of them are the same as our previously annotated baseline. 4) We applied many machine learning and deep learning algorithms to the baseline to find the most accurate one. 5) We improved the performance of the classifiers by measuring the Fleiss Kappa agreement and balancing the dataset accordingly. 6) By filtering only 11.46% of the tweets with a low agreement between raters, we achieved an F1-Score of 0.85 using BERT. Therefore, we conclude that the classifier's performance in hate speech detection on Twitter is related to the agreement between annotators in labeling the data.

In addition to the above experiment, we analyzed the features of tweets with high vs. low agreement to understand what kind of language is easily labeled and what kind is more ambiguous. To perform this task, we focus on the following tweets:

- Tweets labeled unanimously by all five annotators mean the agreement number is 1.
- Tweets with the lowest agreement. We used the threshold < = 0.3 to find these tweets.

We did this analysis on our dataset from phase 2 by making two following sub-datasets:

- Tweets with the complete agreement between annotators, tweets with the agreement 1. There are 758 tweets with agreement 1 in our annotated dataset
- Ambiguous tweets that are tweets with agreement < = 0.3, and there are 135 of them in our dataset

Figures 2D,E, show the distribution of ambiguous tweets and tweets with the full agreement between annotators in three classes. These figures show that fewer hateful tweets are ambiguous, and more ambiguous tweets are from counterhate and neutral classes, which is not a far-fetched argument. Figures 3D,E are wordcloud of these two datasets. They demonstrate that the words racism most common phrases of racismIsVirus, racist, and China, are the most common words in the ambiguous dataset, which means their presence did not help recognize the tweet category. We hope that this study will contribute toward an increased understanding of anti-Asian hate speech on social media, and will help identify and reduce this toxic content.

## Data availability statement

The datasets presented in this article are not readily available because 1- Twitter API does not allow anybody to publish the text of the tweets. 2- upon our IRB approval, we have committed to storing the dataset in a password-protected environment. Requests to access the datasets should be directed to AT, atoliyat@gradcenter.cuny.edu.

## Ethics statement

The studies involving human participants must be reviewed and approved by the IRB (Institutional Review Board) before data collection can begin. For this research, we submitted an IRB application which was accepted by the Human Research Protection Program (HRPP) at City College with approval number #2021-0638. The research participants provided their written informed consent to participate in this study.

## Author contributions

All authors listed have made a substantial, direct, and intellectual contribution to the work and approved it for publication.

## Conflict of interest

The authors declare that the research was conducted in the absence of any commercial or financial relationships that could be construed as a potential conflict of interest.

## Publisher's note

All claims expressed in this article are solely those of the authors and do not necessarily represent those of their affiliated organizations, or those of the publisher, the editors and the reviewers. Any product that may be evaluated in this article, or claim that may be made by its manufacturer, is not guaranteed or endorsed by the publisher.
